# Body temperature correlates with depressive symptom severity: analysis and extension of prior findings in observational cohorts with more than 19,000 participants

**DOI:** 10.3389/fpsyt.2026.1846398

**Published:** 2026-08-19

**Authors:** Severine Soltani, Wendy Hartogensis, Stephan Dilchert, Kyle Flores, Christopher A. Lowry, Charles L. Raison, Frederick M. Hecht, Benjamin L. Smarr, Ashley E. Mason

**Affiliations:** 1 Bioinformatics and Systems Biology Graduate Program, University of California, San Diego, San Diego, CA, United States; 2 Shu Chien-Gene Lay Department of Bioengineering, University of California, San Diego, San Diego, CA, United States; 3 Osher Center for Integrative Health, University of California, San Francisco, San Francisco, CA, United States; 4 Department of Management, Zicklin School of Business, Baruch College, The City University of New York, New York, NY, United States; 5 Halıcıoğlu Data Science Institute, University of California, San Diego, San Diego, CA, United States; 6 Department of Integrative Physiology, University of Colorado Boulder, Boulder, CO, United States; 7 Department of Psychiatry, School of Medicine and Public Health, University of Wisconsin-Madison, Madison, WI, United States

**Keywords:** biomarker, body temperature, depression, skin temperature, wearable device

## Abstract

**Objective:**

Prior studies have reported that higher body temperature correlates with greater depressive symptoms, but did not adjust for potential confounders.

**Methods:**

We used linear regression models and linear mixed models to assess associations between self-reported depressive symptoms and body temperature (once-daily self-reported temperature from a thermometer and minute-level distal temperature from a commercially available wearable device, worn on the finger). Adjusted models covaried for biological sex, age, daylight length, ambient temperature, and number of medical conditions. Distal body temperature metrics included awake and asleep temperatures, the asleep–awake temperature difference, and the diurnal temperature amplitude. Distal body temperature models also covaried for daylight length and ambient temperature over time. Finally, we used adjusted logistic regression models to assess associations between all body temperature metrics and four categories of depressive symptom severity.

**Results:**

In an adjusted linear regression model, the positive association between depressive symptoms and self-reported body temperature remained significant (*b* = 0.940, 95% CI [0.686, 1.193], *p* = 1.7 × 10^–12^). Adjusted associations with awake distal body temperature (*b* = 0.300, 95% CI [0.229, 0.371], *p* = 1.2 × 10^–15^), diurnal distal body temperature amplitude (*b* = -0.204, 95% CI [-0.248, -0.159], *p* = 6.1 × 10^–18^), and asleep-awake distal body temperature difference (*b* = -0.308, 95% CI [-0.375, -0.240], *p* = 6.1 × 10^–18^) were stronger than in unadjusted models. Standardized effect estimates from the adjusted linear regression models indicated that the magnitude of these associations was robust but small in magnitude, with standardized coefficients ranging from -0.067 to 0.061 and partial R² values ranging from 0.0006 to 0.0041. Adjusted linear mixed models using repeated measurements showed similar significant associations between distal body temperature metrics and depressive symptom severity. Adjusted logistic regression models showed that increases in self-reported body temperature and awake distal body temperature conferred significantly higher odds of a more severe depressive symptom category; increases in diurnal distal body temperature amplitude and asleep-awake temperature difference conferred significantly lower odds.

**Conclusion:**

Higher body temperature was associated with increased depressive symptoms, and this association was robust to adjustment for potential confounders. These findings support body temperature as a physiological correlate of depressive symptom severity and motivate further investigation of thermoregulatory measures as part of multimodal approaches for monitoring depressive symptoms.

## 1 Introduction

Depression has emerged as a major public health crisis, with approximately 12% of adults worldwide expected to experience major depressive disorder at some point in their lives (1, 2). The prevalence of depression has sharply increased in recent years, particularly following the onset of the COVID-19 pandemic (3). One key challenge to the diagnosis and treatment of depression is its heterogeneous presentation, which contributes to variable responses across standard interventions (4–6). This clinical and biological heterogeneity complicates the development and evaluation of new therapies (7).

To improve diagnosis and treatment options for depression, previous studies have investigated physiological correlates of depressive symptoms. Among these, alterations to thermoregulatory processes have emerged as a promising candidate biomarker (8). Studies have reported elevated body temperature during both daytime (9) and nighttime (10) among individuals with depression, as well as reduced circadian amplitude of body temperature (11). Identification of such physiological biomarkers may facilitate the delineation of distinct phenotypes of depression and inform the development of more targeted, individualized interventions.

In the largest sample assessed to date, we examined self-reported body temperature and distal body temperature assessed via a wearable sensor in relation to depressive symptoms (12). In particular, we observed that individuals with greater depressive symptom severity had elevated body temperature while awake (i.e., during the daytime), and that each 0.1 °C increase in body temperature significantly increased the risk of having greater depressive symptom severity. Using a commercially available wearable sensor to collect metrics of body temperature, we found that increased distal body temperature while awake and decreased diurnal distal body temperature amplitude were associated with greater depressive symptom severity. When analyzing self-reported body temperature in this previous publication, we accounted for covariates known to potentially affect body temperature, such as age (13), biological sex (13), and time of day (9). Adjusting for these covariates attenuated the association between depressive symptom severity and body temperature; however, this association remained significant. We did not account for any covariates in our analyses exploring depressive symptom severity and sensor-assessed body temperature.

Academic and public responses to the publication were notable for curiosity regarding additional potentially relevant covariates that we had not accounted for in these statistical analyses. Such candidate covariates included (1) medical conditions, (2) daylight length, and (3) outdoor ambient temperature. Each of these covariates have plausible associations with body temperature and/or depression. First, experiencing medical conditions increases risk for depression (14). Additionally, medical conditions, such as diabetes (15) and hypertension (16), may independently affect thermoregulation by reducing blood flow to the skin, which may inhibit heat dissipation from the body’s core. Second, seasons with shorter daylight lengths are associated with increased prevalence of depressive symptoms, as in the case of seasonal affective disorder (17, 18). Finally, prior studies suggest that warmer seasons affect body temperature, though the direction of effect remains unclear with studies showing both increases (19) and decreases (20) in body temperature. Therefore, omitting medical conditions, daylight length, and outdoor ambient temperature as covariates in statistical analyses may bias associations between depressive symptom severity and body temperature.

In the present analyses, we examined the association between body temperature and depressive symptom severity reported in our prior publication after adjusting for (1) local daylight length, (2) local ambient temperature, and (3) number of medical conditions as covariates. Our primary aim was to evaluate the impact of these covariates on self-reported body temperature. We also computed adjusted analyses for the four sensor-assessed distal body temperature metrics we originally reported in Mason et al. (12). We hypothesized that inclusion of additional covariates would attenuate—but not eliminate—an association between self-reported body temperature and depressive symptom severity. We further hypothesized that the sensor-assessed distal body temperature metrics would show smaller yet similar associations with depressive symptoms, consistent with Mason et al. (12).

## 2 Materials and methods

### 2.1 Study overview

The TemPredict Study (21, 22) was a prospective, worldwide, cohort study conducted from March 2020 to November 2020 that continuously collected physiological metrics (e.g., body temperature) using an off-the-shelf wearable device (Oura Ring) with the primary aim of developing an algorithm to identify the onset of COVID-19. Participants also completed baseline, daily, and monthly surveys (Qualtrics Survey Software); participants self-reported their self-collected body temperature in the daily survey. Eligible participants were at least 18 years of age, possessed a smartphone that could pair with the Oura Ring, and could communicate in English. Eligible participants either already possessed an Oura Ring that they used for participation, or the study provided them with an Oura Ring. We recruited participants through invitations delivered within the Oura smartphone app; these invitations contained embedded weblinks for prospective participants to review study details and to begin the study enrollment process (23). All participants provided electronic informed consent. We did not pay participants for their participation. The University of California, San Francisco, Institutional Review Board and the U.S. Department of Defense, Human Research Protection Office approved all study procedures. We followed the Strengthening the Reporting of Observational Studies in Epidemiology reporting guidelines (24).

### 2.2 Measures

#### 2.2.1 Sociodemographic characteristics

Participants reported on these characteristics in the baseline survey. Participants reported their biological sex as “male,” “female,” “other,” or skipped the item. They reported on their biological age in years. They reported any medical conditions on a multiple-choice item wherein they could skip the item or endorse having no medical conditions, hypertension, diabetes (not including pre-diabetes), coronary artery disease or angina, myocardial infarction, congestive heart failure, stroke or transient ischemic attack, atrial fibrillation, sleep apnea, chronic obstructive pulmonary disease (including emphysema or chronic bronchitis), asthma (requiring regular inhaler use), cancer (including leukemia or lymphoma; undergoing active treatment), anemia (or other blood disorder), immunodeficiency (including human immunodeficiency virus), and/or other respiratory issues.

#### 2.2.2 Self-reported depressive symptoms

Participants completed the Patient Reported Outcomes Measurement Information System (PROMIS) Adult Health Profile instrument for depression (v1.0 Form 4a) each month with the recall period modified to reflect the past month rather than the past 7 days in the monthly survey (12, 25, 26). We modified this recall period to better capture overall depressive symptom burden over the monthly assessment period and to reduce participant burden associated with more frequent assessments.

#### 2.2.3 Self-reported body temperature

Participants reported their self-collected body temperature once per day in the daily survey. The daily survey asked participants to report the highest body temperature value they collected within the last day (in °F or °C, per participant preference). For these daily self-reported temperature assessments, participants used their own thermometers (which were not provided by the study, and which may have been of any brand), and we did not ask participants to report the type of thermometer used or the method of body temperature assessment. Qualtrics Survey Software generated a time stamp for each completed survey and also collected the approximate geographical coordinates (latitude and longitude) of the device used to complete the survey.

#### 2.2.4 Sensor-assessed body temperature

Participants wore the Oura Ring Gen2 (27), a commercially available wearable sensor device (Oura Health, Oulu, Finland) on any finger (21). The Oura Ring uses Bluetooth to connect to the Oura App (available in Google Play Store and Apple App Stores). Participants could wear the ring continuously throughout the day and night, and in both wet and dry environments. The Oura Ring assesses distal body temperature using a negative temperature coefficient thermistor (resolution of 0.07 °C) on the internal surface of the device, which is in relatively consistent contact with the skin. The wearable sensor assesses distal (dermal, peripheral) body temperature readings taken from the palm side of the finger base every minute. As described elsewhere (21, 28), the Oura Ring also assesses other physiological metrics, including sleep parameters, heart rate, heart rate variability, respiratory rate, and activity metrics.

### 2.3 Variable preparation

#### 2.3.1 Sociodemographic characteristics and medical conditions

As done in our prior work (12), we created a binary biological sex variable (male and female) and excluded participants who reported their biological sex as “other” or who did not provide a response to the biological sex item. We chose to exclude these participants rather than include them as a separate subgroup because there were very few of these participants (*n* = 17). In addition, “other” and missing responses may reflect a range of heterogeneous circumstances and therefore may not represent a coherent subgroup.

In addition to the sociodemographic variables (see Mason et al. (12)), we computed a medical conditions variable with the following categories: 0 (*no medical conditions*), 1 (*one medical condition*), 2 (*two medical conditions*), and 3 (*three or more medical conditions*). Because nonresponse to the medical condition item was common, excluding participants who did not provide this information would have removed a substantial proportion of the analytic sample and could have introduced selection bias. We therefore retained these participants and coded nonresponse as a separate category rather than treating nonresponse as equivalent to having no medical conditions. We chose to code medical conditions as a graded variable rather than a binary variable (i.e., having no medical conditions or having 1+ medical conditions) as having multiple medical conditions compounds the prevalence of depressive symptoms (29–32). Furthermore, common medical conditions that affect thermoregulation, such as diabetes (15) and hypertension (16), may potentially affect body temperature in an additive manner.

#### 2.3.2 Self-reported depressive symptoms

Each item on the PROMIS depression Form 4a is scored from 1 (*never*) to 5 (*always*), and these item scores are summed to create a total raw summary score. We converted raw PROMIS depression summary scores to T-scores using the conversion tables in the PROMIS scoring manual (33, 34). PROMIS depression T-scores are referenced to a general adult population with a mean score of 50 and standard deviation of 10, with higher T-scores indicating greater depressive symptomatology (26, 35). We used recommended cut points to convert T-scores into categorical depression range variables (mild, moderate, and severe, and “within normal limits” [WNL]) (33, 34). We computed each participant’s average T-score across a maximum of seven monthly PROMIS depression assessments. We then computed the average and standard deviation (*SD*) of all participants’ mean T-scores. We used established thresholds for PROMIS depression T-scores to categorize each participant’s depressive symptom severity based on their mean T-score: WNL (T-score below 55); mild (T-score 55–59.99); moderate (T-score 60–69.99); and severe (T-score 70 or greater).

#### 2.3.3 Self-reported body temperature

We excluded self-reported body temperature data points that were above 38.0 °C, as such data points meet criteria for clinical fever and do not represent baseline physiologic temperatures. We excluded these periods of potential illness because these analyses focus on the association between body temperature and depressive symptoms under usual conditions of daily life.

#### 2.3.4 Body temperature survey time stamp (B_1, 2_)

We derived two variables based on the local time at which participants self-reported their body temperature each day on the daily symptom survey. Specifically, as done by Rausch et al. (9) and in our prior work (12), we computed two variables to account for this time: (B_1_) the cosine of 2*pi*t, where “t” represents the decimal proportion of the day during which the daily survey was completed, and (B_2_) sine of 2*pi*t, for each self-reported body temperature value. As shown in our prior work (12), temperature patterns over time were consistent with the assumption that these body temperatures were reported close to the time of measurement.

#### 2.3.5 Sensor-assessed body temperature

For each participant, we used the 2 weeks of distal body temperature data prior to each completed PROMIS assessment. For each 24-h period, we discarded wearable sensor-assessed distal body temperature values below the 5th percentile value and above the 95th percentile. This served to exclude periods of non-wear and periods of clinical fever and to minimize influence from non-representative extreme values (e.g., outliers), as done in prior research with wearable sensor-assessed metrics (12). Using these filtered distal body temperature data, we calculated four sensor-assessed body temperature metrics:

##### 2.3.5.1 Asleep distal body temperature

Using hypnogram data to determine periods of sleep, we computed the daily average distal body temperature during sleeping minutes.

##### 2.3.5.2 Awake distal body temperature

Using hypnogram data to determine periods of wakefulness, we computed the daily average distal body temperature during minutes awake.

##### 2.3.5.3 Asleep–awake distal body temperature difference

We calculated the difference between the daily average asleep distal body temperature and the daily average awake distal body temperature.

##### 2.3.5.4 Diurnal distal body temperature amplitude

We calculated the daily maximum distal body temperature as the highest daily value and the daily minimum distal body temperature as the lowest daily value for each person and calculated the difference between these two values.

#### 2.3.6 Daylight length

We used each participant’s approximate geographical coordinates (latitude and longitude) collected automatically when they responded to each daily survey to compute local daylight length for each participant. We used the Python package *suntimes* (v1.1.2) (36) to calculate the time difference (in hours) between sunrise and sunset for a given date (day, month, year).

#### 2.3.7 Ambient temperature

We used each participant’s approximate geographical coordinates (latitude and longitude) collected automatically when they responded to each daily survey to compute the daily average ambient temperature for each participant. We used the Python package *meteostat* (v1.6.8) (36) to retrieve daily average ambient temperature data (in °C) from the nearest weather station with available data within a 35,000-meter radius of the participant’s geographical coordinates. If there were no weather stations with ambient temperature measurements within a 35,000-meter radius of the participant’s geographical coordinates, we classified ambient temperature data as missing for that day.

### 2.4 Analytic approach

#### 2.4.1 Software

We performed statistical analyses using Python (v3.12.8) (37, 38) with the *statsmodels* (v0.14.4) (39), *SciPy* (v1.15.1) (40), and *scikit-learn* (v1.6.1) (41) packages.

#### 2.4.2 Data inclusion

We first applied data inclusion criteria identical to those applied in prior work (12). We then created the two adjusted analysis datasets (self-reported body temperature and sensor-assessed body temperature) by applying criteria described in *Variable Preparation*, above. We then used each dataset for its respective unadjusted and adjusted models. Further data inclusion criteria varied by type of analysis. However, for both datasets, we excluded participants who lacked any measurements for either daylight length or ambient temperature variables (*n =* 608 for self-reported body temperature and *n =* 1,557 for sensor-assessed body temperature dataset; see *Ambient temperature*, above).

##### 2.4.2.1 Self-reported body temperature analyses data inclusion

Analyses of self-reported body temperature data included participants who completed one or more PROMIS depression assessments, provided at least one self-reported temperature assessment, and had at least one measurement for both daylight length and ambient temperature.

##### 2.4.2.2 Sensor-assessed body temperature analyses data inclusion

Analyses involving wearable sensor-assessed distal body temperature data included participants who completed one or more PROMIS depression assessments and wore an Oura Ring for at least seven 24-h periods with at least 4 h of asleep and awake distal body temperature data recorded during each 24-h period. As in the self-reported body temperature sample, we required at least one measurement for both daylight length and ambient temperature. We included distal body temperature, daylight length, and ambient temperature data collected in the 2 weeks prior to each completed PROMIS depression assessment. We included these variables from the 2 weeks prior to each completed PROMIS depression assessment to capture physiological measures that were temporally proximal to depressive symptom assessment; recent physiological states may be more closely related to current symptom severity than measurements collected farther in the past.

#### 2.4.3 Unadjusted models

##### 2.4.3.1 Unadjusted linear regression models

We computed an unadjusted linear regression model predicting the average PROMIS depression T-score variable from participants’ (1) average self-reported body temperature, and (2) average of each of the four sensor-assessed distal body temperature metrics. We report unstandardized regression coefficients, standardized regression coefficients, and partial *R*^2^ values for each body temperature metric. Standardized coefficients represent the expected change in the outcome, expressed in standard deviation units, associated with a 1-standard-deviation increase in the predictor, holding all other variables in the model constant.

##### 2.4.3.2 Unadjusted linear mixed models

To preserve time-dependent changes across variables, we computed linear mixed models (LMMs) predicting the monthly PROMIS depression T-score variable from four sensor-assessed distal body temperature metrics (one model per metric), with fixed effects analogous to those in the linear regression models and adding a random intercept to account for the correlation of repeated measures. We averaged sensor-assessed distal body temperature metric measurements from the 2 weeks prior to each completed PROMIS depression assessment.

##### 2.4.3.3 Unadjusted logistic regression models

We computed separate unadjusted logistic regression models for each categorical depressive symptom range on the PROMIS measure (*mild*, *moderate*, and *severe*, each with *WNL* as the reference category) as outcomes and (1) self-reported body temperature and (2) four sensor-assessed distal body temperature metrics (in separate models for each metric), as predictors. We used established thresholds for PROMIS depression T-scores, with separate models for mild vs. WNL; moderate vs. WNL; and severe vs. WNL, as the outcome variable, and self-reported body temperature as the predictor, scaled per 0.1 °C (26). We report odds ratios (ORs) with 95% confidence intervals (CIs) for the odds for each depressive symptom range with a 0.1 °C increase in average self-reported body temperature and visualized this in a forest plot.

#### 2.4.4 Adjusted models

Adjusted models included the following covariates: age, biological sex, body temperature survey time stamp (self-reported body temperature analyses only), daylight length, ambient temperature, and number of medical conditions.

##### 2.4.4.1 Adjusted linear regression models

We computed multiple linear regression models predicting the average PROMIS depression T-score variable from (1) self-reported body temperature (one model), and (2) four sensor-assessed distal body temperature metrics (one model per metric) and covariates. Survey timestamp data were not relevant or included in the sensor-assessed models, as the metrics are continuously measured.

##### 2.4.4.2 Adjusted linear mixed models

To preserve time-dependent changes across variables, we computed LMMs predicting the monthly PROMIS depression T-score variable from four sensor-assessed distal body temperature metrics (one model per metric) and covariates, with fixed effects analogous to those in the linear regression models and adding a random effect to account for the correlation of repeated measures. We averaged sensor-assessed distal body temperature metric measurements from the 2 weeks prior to each completed PROMIS depression assessment.

##### 2.4.4.3 Adjusted logistic regression models

We computed logistic regression models predicting depressive symptom category variables (mild, moderate, and severe, each with WNL as the reference category) from (1) self-reported body temperature (one model), and (2) four sensor-assessed distal body temperature metrics (one model for each metric) and covariates.

#### 2.4.5 Receiver operating characteristic comparisons

We computed ROC curve analyses for each logistic regression model and calculated the area under the curve (AUC) to evaluate how well body temperature metrics could identify depressive symptoms in three comparisons (WNL vs. mild; WNL vs. moderate; and WNL vs. severe) across unadjusted and adjusted models. We used Youden’s Index to identify body temperature thresholds to evaluate sensitivity and specificity based on those threshold values (42). Youden’s index can be calculated as (sensitivity + specificity – 1) and ranges between 1 and 0, with 1 indicating perfect sensitivity and specificity and 0 indicating results no better than chance (42). Maximizing Youden’s index in ROC analysis identifies the value of a continuous predictor that best distinguishes cases and non-cases, and is equivalent to locating the point on the curve that is furthest from the line of chance (43).

#### 2.4.6 Adjustment for multiple comparisons

To account for multiple comparisons, we controlled the false discovery rate at 5% using the Benjamini-Hochberg method applied separately to models involving (1) body temperature and (2) sensor-assessed distal body temperature. Reported *p*-values in the main manuscript are false-discovery rate adjusted; values below 0.05 were considered significant. No correction for multiple comparisons was applied to analyses presented in the **Supplementary Materials**.

## 3 Results

### 3.1 Descriptive information

#### 3.1.1 Self-reported body temperature dataset

After applying the above-described variable preparations, the self-reported body temperature dataset included 20,255 participants. Participants’ mean age (*SD*) was 47.0 (12.6) years; they were 53% male and 47% female; and their mean PROMIS depression T-score (*SD*) was 51.49 (overall *SD* = 7.37; within-person *SD* = 2.12), which is within normal limits (WNL). On average, participants completed 3.6 of 7 possible PROMIS depression assessments and self-reported a total of 559,237 body temperature assessments (mean of 27.5 daily temperature reports per participant).

Of these participants, 13,186 had an average depressive symptom severity WNL (T-score < 55), 4,395 had an average depressive symptom severity within the mild range (T-score ≥ 55 and < 60), 2,583 had an average depressive symptom severity within the moderate range (T-score ≥ 60 and < 70), and 91 had an average depressive symptom severity within the severe range (T-score ≥ 70) of the PROMIS depression assessment (44). The average range between participants’ lowest to highest scores was 5.25 (*SD* = 5.50), and the median range was 3.90 (interquartile range [*IQR*] = 0–8.3). This dataset included a geographically diverse set of individuals from 102 different countries.

#### 3.1.2 Sensor-assessed distal body temperature dataset

After applying the above-described variable preparations, the sensor-assessed body temperature dataset included 19,507 participants. Participants’ mean age (*SD*) was 46.6 (12.1) years; they were 56% male and 44% female; and their mean PROMIS depression T-score (*SD*) was 50.92 (overall *SD* = 7.25; within-person *SD* = 2.13), which is WNL. On average, participants completed 3.6 of 7 possible PROMIS depression assessments and had an average of 36.2 days of distal body temperature data with at least (1) 4 h of distal body temperature data during the asleep period and (2) at least 4 h of distal body temperature data during the awake period for each 24-h period available within our timeframe of analyses.

Of these participants, 13,297 had an average depressive symptom severity WNL (T-score < 55), 3,978 had an average depressive symptom severity within the mild range (T-score ≥ 55 and < 60), 2,166 had an average depressive symptom severity within the moderate range (T-score ≥ 60 and < 70), and 66 had an average depressive symptom severity within the severe range (T-score ≥ 70) of the PROMIS depression assessment (44). The average range between participants’ lowest to highest scores was 5.24 (*SD* = 5.50), and the median range was 3.90 (*IQR* = 0–8.3). This dataset included a geographically diverse set of individuals from 98 different countries.

### 3.2 Self-reported body temperature

#### 3.2.1 Unadjusted linear regression models

Greater daily self-reported body temperature was significantly associated with greater average PROMIS depression T-scores (*b =* 1.749, 95% CI [1.490, 2.008]; **Table 1**); this coefficient corresponds to an estimated 1.749-point increase in PROMIS depression T-score per 1 °C increase in self-reported body temperature, or rather a 0.175-point increase in PROMIS depression T-score per a more biologically plausible 0.1 °C increase in self-reported body temperature.

**Table 1:** Unadjusted and adjusted linear models regressing average PROMIS depression T-scores onto self-reported body temperature and wearable sensor-assessed body temperature metrics.

Variable	Unadjusted			Adjusted		
	Regression coefficient [95% CI]	Standardized regression coefficient [95% CI]	Partial *R*^2^	Regression coefficient [95% CI]	Standardized regression coefficient [95% CI]	Partial *R*^2^
Self-reported body temperature	1.749*** [1.490, 2.008]	0.093*** [0.079, 0.106]	0.0086	0.940*** [0.686, 1.193]	0.050*** [0.036, 0.063]	0.0026
Asleep distal body temperature	0.909*** [0.699, 1.119]	0.061*** [0.047, 0.075]	0.0037	-0.405*** [-0.628, -0.181]	-0.027*** [-0.042, -0.012]	0.0006
Awake distal body temperature	0.090* [0.021, 0.159]	0.018* [0.004, 0.032]	0.0003	0.300*** [0.229, 0.371]	0.061*** [0.046, 0.075]	0.0035
Asleep–awake distal body temperature difference	0.008 [-0.056, 0.072]	0.002 [-0.012, 0.016]	<0.0001	-0.308*** [-0.375, -0.240]	-0.067*** [-0.082, -0.053]	0.0041
Diurnal distal body temperature amplitude	-0.049* [-0.092, -0.006]	-0.016* [-0.030, -0.002]	0.0003	-0.204*** [-0.248, -0.159]	-0.067*** [-0.082, -0.052]	0.0041

CI, confidence interval. *p < 0.05, **p < 0.01, ***p < 0.001. *P*-values are false discovery rate adjusted at 5% using the Benjamini-Hochberg method.

#### 3.2.2 Adjusted linear regression models

Greater daily self-reported body temperature was associated with greater average PROMIS depression T-scores after accounting for the above-described covariates (*b =* 0.940, 95% CI [0.686, 1.193]; **Table 1**). Neither ambient temperature nor daylight length was associated with PROMIS depression T-scores (**Table 2**). Individuals with a greater number of medical conditions had significantly higher PROMIS depression T-scores relative to those without any medical conditions (1 condition: *b =* 0.917, 95% CI [0.519, 1.314]; 2 conditions: *b =* 1.448, 95% CI [0.854, 2.041]; 3+ conditions: *b =* 2.446, 95% CI [1.592, 3.300]; **Table 2**).

**Table 2:** Adjusted linear model (incorporating self-reported body temperature) regressing average PROMIS depression T-scores onto ambient temperature, daylight length, and number of medical conditions.

Variable	Regression coefficient [95% CI]	*p*-value
Daylight length (h)	-0.061 [-0.139, 0.017]	0.14
Ambient temperature (°C)	-0.017 [-0.034, 0.000]	0.061
No medical conditions^R^		
Medical conditions not reported	0.023 [-0.239, 0.286]	0.86
1 medical condition	0.917 [0.519, 1.314]	< 0.001
2 medical conditions	1.448 [0.854, 2.041]	< 0.001
3+ medical conditions	2.446 [1.592, 3.300]	< 0.001

CI, confidence interval; ^R^, reference group. *P*-values shown are false discovery rate adjusted at 5% using the Benjamini-Hochberg method.

#### 3.2.3 Unadjusted logistic regression models

**Figure 1:**
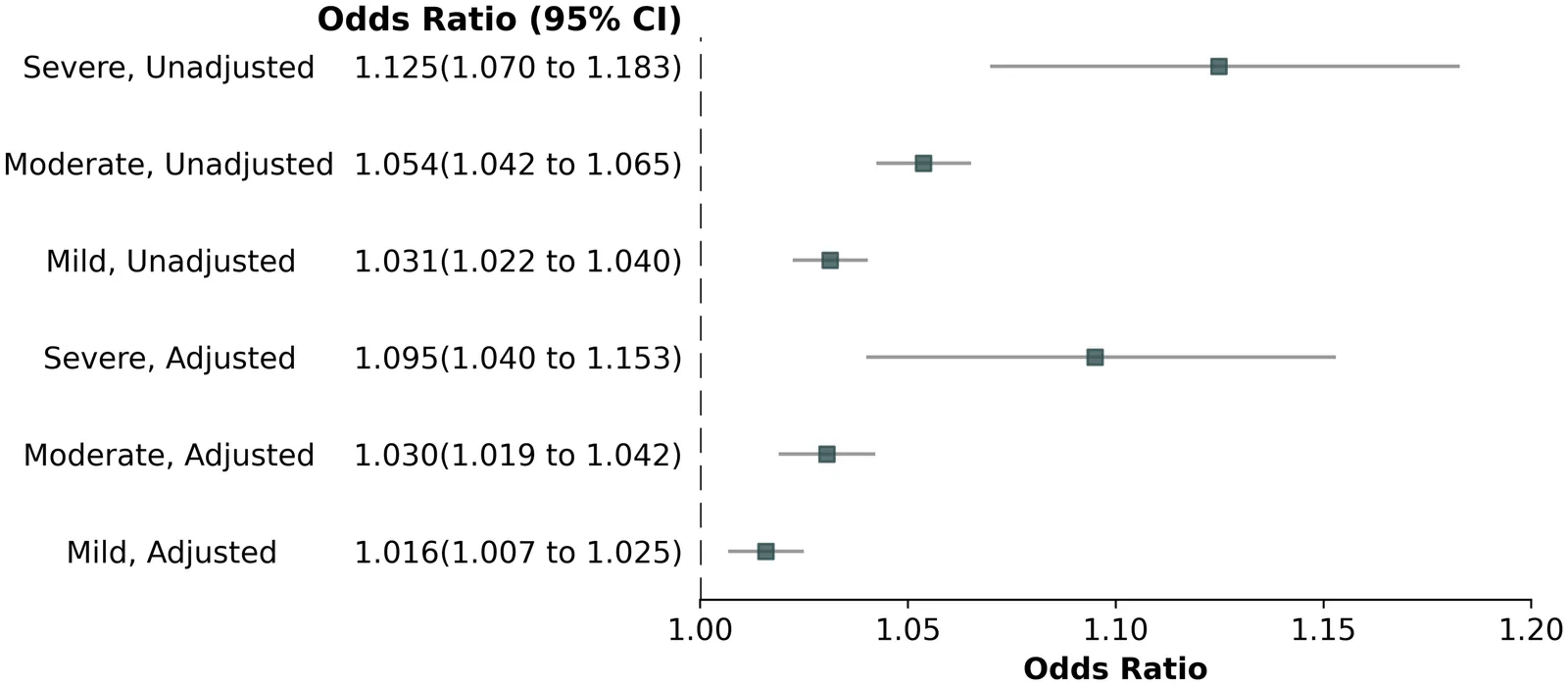
Forest plot depicting odds ratios from logistic regression models using self-reported body temperature measurements. Adjusted and unadjusted models predict PROMIS depression T-score categories (mild, moderate, severe) vs. depressive symptoms within normal limits (WNL) from self-reported body temperature (scaled per 0.1 °C).

Higher self-reported body temperature was associated with significantly increased odds of having PROMIS depression T-scores within the mild (OR = 1.031, 95% CI [1.022, 1.040]), moderate (OR = 1.054, 95% CI [1.042, 1.065]), and severe (OR = 1.125, 95% CI [1.070, 1.183]) depressive symptom ranges relative to WNL (**Table 3**, **Figure 1**). Meaning, for example, that each 0.1 °C increase in self-reported body temperature was associated with approximately a 12.5% increase in the odds of severe depressive symptoms compared with WNL.

**Table 3:** Unadjusted and adjusted logistic models regressing PROMIS depression T-score categories (mild, moderate, severe, with T-scores within normal limits [WNL] as the reference range) onto average self-reported body temperature and wearable sensor-assessed body temperature metrics (scaled per 0.1 °C).

Comparison	Variable	Unadjusted		Adjusted	
		Odds ratio [95% CI]	*p*-value	Odds ratio [95% CI]	*p*-value
Mild vs. WNL	Self-reported body temperature	1.031 [1.022, 1.040]	< 0.001	1.016 [1.007, 1.025]	< 0.001
	Asleep distal body temperature	1.017 [1.009, 1.024]	< 0.001	0.989 [0.981, 0.997]	0.013
	Awake distal body temperature	1.002 [0.999, 1.004]	0.25	1.006 [1.003, 1.009]	< 0.001
	Asleep–awake distal body temperature difference	1.000 [0.998, 1.002]	0.84	0.994 [0.991, 0.996]	< 0.001
	Diurnal distal body temperature amplitude	0.999 [0.998, 1.000]	0.21	0.996 [0.994, 0.997]	< 0.001
Moderate vs. WNL	Self-reported body temperature	1.054 [1.042, 1.065]	< 0.001	1.030 [1.019, 1.042]	< 0.001
	Asleep distal body temperature	1.025 [1.015, 1.035]	< 0.001	0.982 [0.972, 0.992]	0.0012
	Awake distal body temperature	1.006 [1.003, 1.009]	< 0.001	1.013 [1.009, 1.016]	< 0.001
	Asleep–awake distal body temperature difference	0.997 [0.994, 1.000]	0.083	0.987 [0.984, 0.990]	< 0.001
	Diurnal distal body temperature amplitude	0.996 [0.994, 0.998]	< 0.001	0.991 [0.989, 0.993]	< 0.001
Severe vs. WNL	Self-reported body temperature	1.125 [1.070, 1.183]	< 0.001	1.095 [1.040, 1.153]	< 0.001
	Asleep distal body temperature	1.025 [0.972, 1.080]	0.38	0.971 [0.920, 1.025]	0.32
	Awake distal body temperature	1.022 [1.003, 1.041]	0.031	1.030 [1.010, 1.050]	0.0043
	Asleep–awake distal body temperature difference	0.985 [0.969, 1.001]	0.083	0.970 [0.952, 0.989]	0.0025
	Diurnal distal body temperature amplitude	0.988 [0.977, 0.998]	0.026	0.982 [0.971, 0.993]	0.0025

CI, confidence interval. *P*-values shown are false discovery rate adjusted at 5% using the Benjamini-Hochberg method.

#### 3.2.4 Adjusted logistic regression models

Adjusting for covariates attenuated differences between participants WNL and other categorical depressive symptom ranges, but higher self-reported body temperature remained significantly associated with increased odds of having PROMIS depression T-scores within the mild (OR = 1.016, 95% CI [1.007, 1.025]), moderate (OR = 1.030, 95% CI [1.019, 1.042]), and severe (OR = 1.095, 95% CI [1.040, 1.153]) ranges relative to WNL (**Table 3**, **Figure 1**).

#### 3.2.5 Unadjusted vs. adjusted ROC curve analyses

**Figure 2:**
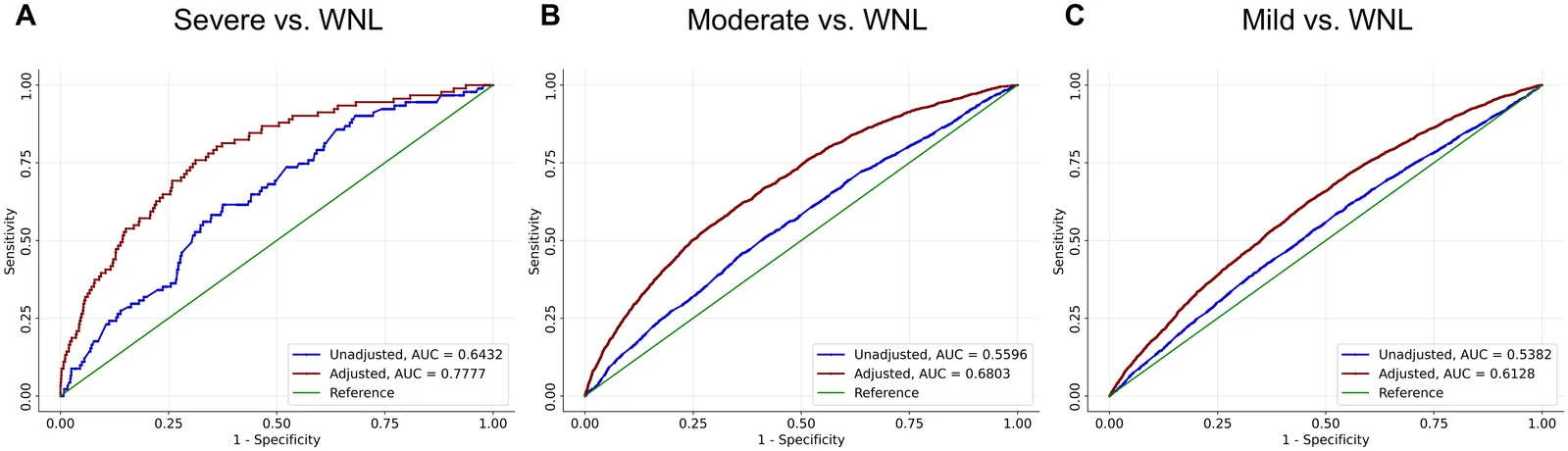
Receiver operating characteristic (ROC) analyses for self-reported body temperature measurements. Figure panel **(A)** depicts ROC curves based on unadjusted and adjusted logistic regression models for PROMIS depression T-scores in the severe range, the **(B)** moderate range, and **(C)** the mild range, versus PROMIS depression T-scores within normal limits (WNL).

Adjusted logistic regression models demonstrated better discernment of all depressive symptom ranges vs. WNL relative to unadjusted models (**Supplementary Tables 1**, **2**, **Figure 2**). AUCs were consistently higher in adjusted models relative to unadjusted models. Both sensitivity and specificity were highest for detecting severe depressive symptoms in adjusted models (75.82% and 68.77%, respectively). In unadjusted models, sensitivity remained highest (61.54%) for detecting PROMIS depression T-scores in the severe range, whereas specificity was highest (63.57%) for detecting PROMIS depression T-scores in the moderate range.

### 3.3 Sensor-assessed body temperature

#### 3.3.1 Unadjusted linear regression models

In unadjusted linear regression models, greater asleep (*b =* 0.909, 95% CI [0.699, 1.119]) and awake (*b =* 0.090, 95% CI [0.021, 0.159]) distal body temperature were significantly associated with greater PROMIS depression T-scores. Asleep–awake temperature difference showed no association, and diurnal amplitude was negatively associated (*b =* -0.049, 95% CI [-0.092, -0.006]) with PROMIS depression T-score (**Table 1**).

#### 3.3.2 Adjusted linear regression models

In adjusted linear regression models, the magnitude of the coefficients tended to increase after statistically accounting for age, biological sex, daylight length, ambient temperature, and number of medical conditions (**Table 1**, **Supplementary Table 3**). Notably, asleep distal body temperature was negatively associated with PROMIS depression T-score (*b =* -0.405, 95% CI [-0.628, -0.181]). Awake distal body temperature remained significantly positively associated with depressive symptom severity (*b =* 0.300, 95% CI [0.229, 0.371]). Both asleep–awake difference (*b =* -0.308, 95% CI [-0.375, -0.240]) and diurnal amplitude (*b =* -0.204, 95% CI [-0.248, -0.159]) were significantly negatively associated with greater depressive symptom severity (**Table 1**). Ambient temperature was significantly negatively associated with depressive symptom severity in the models incorporating asleep–awake difference (*b =* -0.022, 95% CI [-0.042, -0.001]) and diurnal amplitude (*b =* -0.023, 95% CI [-0.043, -0.002]) but was not significantly associated with depressive symptom severity in models incorporating asleep or awake distal body temperature (**Supplementary Table 3**). For all distal body temperature metrics, individuals with a greater number of medical conditions had significantly higher PROMIS depression T-scores relative to those without any medical conditions (**Supplementary Table 3**).

#### 3.3.3 Unadjusted linear mixed models

In unadjusted LMMs, asleep distal body temperature remained positively associated with PROMIS depression T-scores (*b =* 0.691, 95% CI [0.548, 0.835]) whereas awake distal body temperature was negatively associated with PROMIS depression T-scores (*b =* -0.105, 95% CI [-0.147, -0.064]). Both asleep–awake distal body temperature difference (*b =* 0.145, 95% CI [0.106, 0.185]) and diurnal distal body temperature amplitude (*b =* 0.088, 95% CI [0.059, 0.118]) were significantly positively associated with PROMIS depression T-scores (**Table 4**).

**Table 4:** Unadjusted and adjusted linear mixed models regressing monthly PROMIS depression T-scores onto average wearable sensor-assessed body temperature metrics from 2 weeks prior to the PROMIS survey date.

Variable	Unadjusted		Adjusted	
	Regression coefficient [95% CI]	*p*-value	Regression coefficient [95% CI]	*p*-value
Asleep distal body temperature	0.691 [0.548, 0.835]	< 0.001	-0.169 [-0.318, -0.020]	0.032
Awake distal body temperature	-0.105 [-0.147, -0.064]	< 0.001	0.171 [0.124, 0.218]	< 0.001
Asleep–awake distal body temperature difference	0.145 [0.106, 0.185]	< 0.001	-0.173 [-0.219, -0.128]	< 0.001
Diurnal distal body temperature amplitude	0.088 [0.059, 0.118]	< 0.001	-0.132 [-0.166, -0.098]	< 0.001

CI, confidence interval. *P*-values shown are false discovery rate adjusted at 5% using the Benjamini-Hochberg method.

#### 3.3.4 Adjusted linear mixed models

The adjusted LMMs (**Table 4**, **Supplementary Table 4**) yielded largely similar, though attenuated, associations with PROMIS depression T-scores compared to the final adjusted linear regression models (**Table 1**). As in the adjusted linear regression model, greater asleep distal body temperature was notably significantly negatively associated with PROMIS depression T-score (*b =* -0.169, 95% CI [-0.318, -0.020]) in a model adjusted for age, biological sex, ambient temperature, daylength, and number of medical conditions. The associations between depressive symptom severity and each of the remaining three distal body temperature metrics were also consistent with adjusted linear regression models and statistically significant: awake distal body temperature was positively associated with depressive symptom severity (*b =* 0.171, 95% CI [0.124, 0.218]) whereas the asleep–awake difference (*b =* -0.173, 95% CI [-0.219, -0.128]) and diurnal amplitude (*b =* -0.132, 95% CI [-0.166, -0.098]) were negatively associated with depressive symptom severity.

#### 3.3.5 Unadjusted logistic regression models

**Figure 3:**
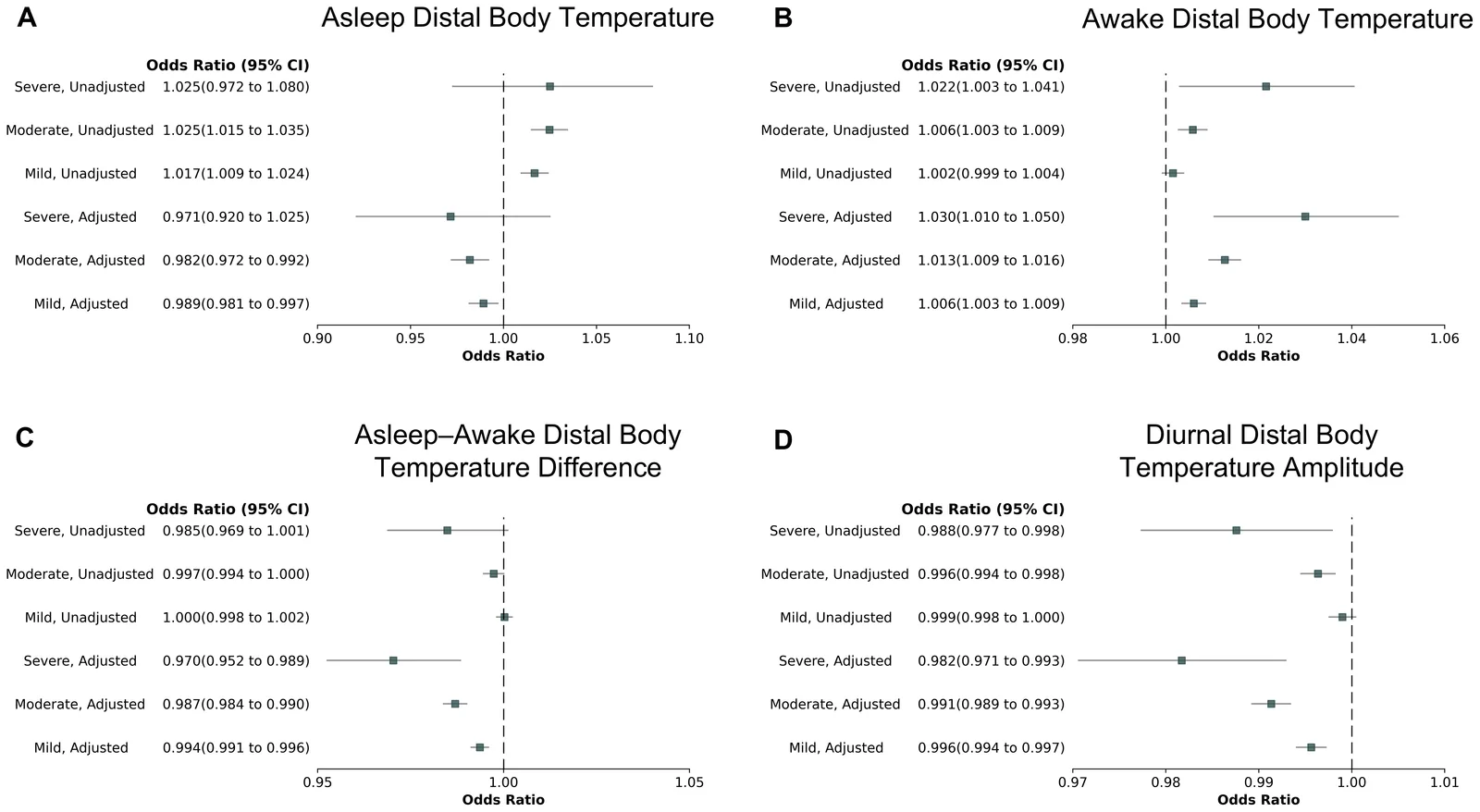
Forest plot depicting odds ratios from logistic regression models using sensor-assessed distal body temperature metrics. Adjusted and unadjusted models predict PROMIS depression T-score categories (mild, moderate, severe) vs. depressive symptom severity within normal limits (WNL) from each sensor-assessed distal body temperature metric (scaled per 0.1 °C) for **(A)** asleep distal body temperature, **(B)** awake distal body temperature, **(C)** asleep–awake distal body temperature difference, and **(D)** diurnal distal body temperature amplitude. .

We report unadjusted logistic regression models individually for each distal body temperature metric: (1) asleep distal body temperature, (2) awake distal body temperature, (3) asleep–awake distal body temperature difference, and (4) diurnal distal body temperature amplitude (**Table 3**, **Figure 3**).

##### 3.3.5.1 Asleep distal body temperature

Each 0.1 °C increase in asleep distal body temperature was associated with significantly increased odds of having average PROMIS depression T-scores within the mild (OR = 1.017, 95% CI [1.009, 1.024]) and moderate (OR = 1.025, 95% CI [1.015, 1.035]) depressive symptom ranges relative to WNL. For average PROMIS depression T-scores within the severe range vs. WNL, the increased odds of depressive symptoms did not reach statistical significance.

##### 3.3.5.2 Awake distal body temperature

Each 0.1 °C increase in awake distal body temperature was associated with significantly increased odds of having PROMIS depression T-scores within the moderate (OR = 1.006, 95% CI [1.003, 1.009]) and severe (OR = 1.022, 95% CI [1.003, 1.041]) depressive symptom ranges relative to WNL. No significant association was observed for the mild depressive symptom range vs. WNL.

##### 3.3.5.3 Asleep–awake distal body temperature difference

No significant associations were observed between increases in asleep–awake distal body temperature difference and any depressive symptom severity range relative to WNL. However, odds ratios trended in the inverse direction, with progressively lower odds of depressive symptoms per 0.1 °C increase in asleep–awake distal body temperature difference as depressive symptom severity increased.

##### 3.3.5.4 Diurnal distal body temperature amplitude

Similar to asleep–awake distal body temperature difference, each 0.1 °C increase in diurnal distal body temperature amplitude was inversely associated with the odds of average PROMIS depression T-scores within the moderate (OR = 0.996, 95% CI [0.994, 0.998]) and severe (OR = 0.988, 95% CI [0.977, 0.998]) depressive symptom ranges relative to WNL. There was not a significant decrease in odds of having PROMIS depression T-scores within the mild range vs. WNL.

#### 3.3.6 Adjusted logistic regression models

We report adjusted logistic regression models individually for each body temperature metric: (1) asleep distal body temperature, (2) awake distal body temperature, (3) asleep–awake distal body temperature difference, and (4) diurnal distal body temperature amplitude (**Table 3**, **Figure 3**).

##### 3.3.6.1 Asleep distal body temperature

In contrast with the unadjusted model, each 0.1 °C increase in asleep distal body temperature was associated with significantly decreased odds of having average PROMIS depression T-scores within the mild (OR = 0.989, 95% CI [0.981, 0.997]) and moderate (OR = 0.982, 95% CI [0.972, 0.992]) depressive symptom ranges relative to WNL. For average PROMIS depression T-scores within the severe range vs. WNL, the odds ratio was lower than for both mild and moderate vs. WNL (OR = 0.971, 95% CI [0.920, 1.025]) but did not reach statistical significance. The inclusion of age and biological sex as covariates contributed to the reversal in direction of association, each of which independently shifted the odds ratios toward decreased odds of depressive symptoms with each 0.1 °C increase in asleep distal body temperature.

##### 3.3.6.2 Awake distal body temperature

Each 0.1 °C increase in awake distal body temperature was significantly associated with increased odds of having PROMIS depression T-scores within the mild (OR = 1.006, 95% CI [1.003, 1.009]), moderate (OR = 1.013, 95% CI [1.009, 1.016]), and severe (OR = 1.030, 95% CI [1.010, 1.050]) depressive symptom ranges relative to WNL. We observed increases in the odds ratios as depressive symptom severity increased.

##### 3.3.6.3 Asleep–awake distal body temperature difference

Each 0.1 °C increase in asleep–awake distal body temperature difference was significantly associated with lower odds of having PROMIS depression T-scores within the mild (OR = 0.994, 95% CI [0.991, 0.996]), moderate (OR = 0.987, 95% CI [0.984, 0.990]), and severe (OR = 0.970, 95% CI [0.952, 0.989]) depressive symptom ranges relative to WNL. We observed decreased odds ratios as depressive symptom severity increased.

##### 3.3.6.4 Diurnal distal body temperature amplitude

Each 0.1 °C increase in diurnal distal body temperature amplitude was significantly associated with lower odds of having PROMIS depression T-scores within the mild (OR = 0.996, 95% CI [0.994, 0.997]), moderate (OR = 0.991, 95% CI [0.989, 0.993]), and severe (OR = 0.982, 95% CI [0.971, 0.993]) depressive symptom ranges relative to WNL. We observed decreases in the odds ratios as depressive symptom severity increased.

#### 3.3.7 Unadjusted vs. adjusted ROC curve analyses

Across all sensor-assessed body temperature metrics, adjusted logistic regression models demonstrated better discernment of all categorical depressive symptom ranges vs. WNL relative to unadjusted models based on AUCs (**Supplementary Tables 1**, **2**, **Supplementary Figure 1**): AUCs for unadjusted models ranged from 0.501 to 0.598 while AUCs for adjusted models ranged from 0.607 to 0.793. Among adjusted models, sensitivity was generally highest for detecting severe depressive symptoms (sensitivity range = 63.64%–68.18%). Unadjusted models showed limited ability to discern between categorical depressive symptom ranges vs. WNL, particularly for the mild depressive symptom range (AUC range = 0.501-0.526; sensitivity range = 18.45%–28.08%).

## 4 Discussion

This study expands on previously reported associations between depressive symptom severity and sensor-assessed body temperature by accounting for covariates with established associations with body temperature, depressive symptoms, or both. We observed that the association between elevated self-reported body temperature and greater depressive symptom severity in Mason et al. (12) remained robust despite adjusting for additional covariates, including daylight length, ambient temperature, and number of medical conditions. Greater sensor-assessed awake distal body temperature was significantly associated with greater depressive symptom severity. The remaining distal body temperature metrics (i.e., asleep, asleep-wake difference, diurnal amplitude) were negatively associated with greater depressive symptom severity. Altogether, these findings further underscore associations between body temperature and depressive symptom severity.

The observed negative associations between depressive symptom severity and each asleep–awake difference and diurnal amplitude align with prior literature showing negative associations between these metrics and depressive symptoms (11, 45–47). More recently, studies assessing body temperature using wearable devices have emerged. However, there are few studies assessing the association between continuously measured distal body temperature and depressive symptoms, and many of the findings from these studies are not consistent. Lorenz et al. (48) found that participants with depressive symptoms had lower skin temperature amplitude, which aligns with the smaller distal temperature amplitude observed in the present study. Another study by McDuff et al. (49) found overall higher distal body temperature, seemingly due to higher daytime distal body temperatures, among participants with elevated depressive symptoms; they also found higher tonic electrodermal activity, which is weakly correlated with distal body temperature (50). Diverging from the present study, a recent study found lower overall distal body temperatures among participants reporting anxiety symptoms and depressive disorder symptoms, seemingly spanning across both daytime and nighttime (51). Interestingly, another study found a notably *higher* distal body temperature amplitude (though, not significantly) with *lower* daytime distal body temperatures (52). Finally, a third study found that among older adults, higher distal body temperature predicted milder depressive symptoms (53). These mixed findings may reflect differences not limited to the wearable devices used, medication use, comorbid medical conditions, and the population being studied. Although wearable devices enable continuous biometric monitoring under free-living conditions, these settings provide less experimental control over environmental and behavioral factors that may influence the measurements.

Other wearable device metrics, such as activity and sleep-wake patterns, have more consistent associations with depressive symptoms. Briefly, lower amplitude of activity was associated with increased lifetime risk of major depressive disorder in a study of over 91,000 participants (54). Another study found participants with depressive symptoms had both lower overall activity and amplitude of activity (55). Multiple studies found greater sleep fragmentation among those with depressive symptoms (55, 56). Emerging literature suggests that the consistency of sleep-wake patterns may influence one’s risk of developing depressive symptoms independent of sleep duration (57). Many existing studies have combined these measures with other wearable-derived metrics (e.g., heart rate, heart rate variability, blood oxygen saturation, and sleep stages) in algorithms designed to classify individuals by depressive symptom severity and characterize associated physiological patterns (53, 58–60). The present study contributes to this broader effort by characterizing body temperature metrics as additional wearable-derived metrics that may complement activity, sleep, and other frequently utilized metrics in assessments of depressive symptom severity.

Although the associations between body temperature metrics and depressive symptom severity were statistically significant, the magnitude of these associations was relatively small when evaluated using standardized effect estimates. Standardized linear regression coefficients and partial *R*^2^ values indicated that the observed associations were small in standardized terms, with adjusted standardized coefficients ranging from -0.067 to 0.061 and partial *R*^2^ values ranging from 0.0006 to 0.0041. Confidence intervals for these estimates consistently excluded the null, indicating that the associations were estimated with relatively high precision despite their small magnitude. Thus, while body temperature metrics were associated with depressive symptom severity after adjustment for relevant covariates, they explained only a small proportion of the variability in depressive symptoms beyond established covariates including demographics, environment, and medical conditions. These findings do not suggest that body temperature alone is sufficient as a clinical biomarker of depressive symptoms; rather, they support the potential value of thermoregulatory metrics as one component of a broader, multimodal approach to characterizing or monitoring depressive symptom severity.

These analyses expand on our prior reports (12) of associations between sensor-assessed body temperature and depressive symptom severity. Unadjusted and adjusted analyses of awake distal body temperature reported here replicated our prior finding that awake distal body temperature was higher in participants belonging to more severe depressive symptom categories. In our prior unadjusted analyses, we found that both asleep–awake distal body temperature difference and diurnal distal body temperature amplitude were smaller in participants belonging to more severe depressive symptom categories. Adjusting for relevant covariates aligned with our prior report: we found that smaller asleep–awake distal body temperature difference and diurnal distal body temperature amplitude were significantly associated with greater depressive symptom severity. These findings align with prior studies reporting negative associations between these metrics and depressive symptom severity (11, 45, 47). Altogether, our findings extend our and others’ earlier work and provide further support for observed associations between depressive symptom severity and changes in distal body temperature.

One notable finding was that ROC curve analyses demonstrated greater discrimination for severe depressive symptoms compared with mild depressive symptoms despite the smaller number of participants in the severe symptom group. This difference may reflect the fact that ROC performance depends primarily on the degree of separation between the distributions of body temperature metrics across groups rather than sample size alone. Participants with more severe depressive symptoms may have more pronounced or consistent alterations in thermoregulatory processes, whereas mild depressive symptoms may represent a more heterogeneous group with greater overlap in physiological characteristics with individuals without clinically significant symptoms (i.e., WNL). However, given the smaller group of participants with severe depressive symptoms, these estimates may be less precise and should be interpreted cautiously.

Another noteworthy finding was that asleep distal body temperature was negatively associated with depressive symptom severity after adjusting for covariates. In our prior unadjusted analyses (12) and unadjusted analyses here, this association was in the reverse direction. Notably, prior studies have linked greater asleep *core* body temperature with greater depressive symptoms (10, 45, 47). We believe that the adjusted analyses reported here may partially reflect a known process of body temperature regulation: i.e., during sleep, core body temperature decreases while distal temperature increases (reflecting heat loss via the periphery) (61). However, the reversal in the association between asleep distal body temperature and depressive symptom severity after covariate adjustment is not fully explained by differences between distal and core body temperature measures. One potential explanation is that age- and sex-related differences in thermoregulation influence the relationship between asleep distal temperature and depressive symptoms: the prevalence of depression is known to vary across age (62) and between biological sexes (63), so accounting for these covariates provides a more accurate estimate of the association between asleep distal body temperature and depressive symptom severity. Importantly, sex differences in body temperature may play a role in this reversal in association. Although overall sex differences in distal body temperature are mixed, distal body temperature varies across the female lifespan, including across hormonal states. Prior studies have demonstrated higher distal body temperatures during the luteal compared with follicular phase of the menstrual cycle during sleep (64, 65), alterations associated with contraceptive use (66), and increased distal temperature with altered rhythmicity during pregnancy (28). Additionally, postmenopausal women exhibit differences in distal body temperature rhythms compared with younger women, including reduced distal body temperature rhythm amplitude and earlier timing of the daily temperature peak, suggesting age-related changes in peripheral thermoregulatory regulation (67). Because age and biological sex were included as covariates in the adjusted models, accounting for these demographic factors may have altered the estimated association between asleep distal temperature and depressive symptom severity. Because information on reproductive aging and related physiological changes was not available in the present cohort, future studies incorporating these measures with longitudinal assessments of distal body temperature rhythms are required to further characterize the association between distal body temperature and depressive symptoms.

There are several possible reasons that adjusting for known covariates of depressive symptoms and/or body temperature did not nullify the associations observed in Mason et al. (12). Both ambient temperature (68–70) and daylight length (17, 18, 71) have been associated with depressive symptom severity, which motivated their inclusion as covariates. To remain consistent with the analytic approach used in Mason et al. (12), we conducted an adjusted linear regression model that aggregated ambient temperature and daylight length across the study period (i.e., spring to fall of 2020) for each participant. This aggregation likely averaged out the effect of these time-dependent variables, potentially diminishing their effect on depressive symptom severity and self-reported body temperature. Previous studies suggest that higher ambient temperature may have a smaller impact on core body temperature relative to distal body temperature (72), which was consistent with our findings. Nevertheless, participants likely spent most of their time in temperature-regulated environments (e.g., home, cars, or stores (73);), minimizing exposure to low or high enough ambient temperatures for sufficient durations of time to significantly impact body temperature. Furthermore, the design of the wearable device limits the potential for environmental factors to bias body temperature assessment, as the sensor is embedded on the inner surface of the ring (against the skin). For example, previous studies have used the Oura Ring to assess changes in distal body temperature associated with the menstrual cycle (65, 74), suggesting that the temperature measurements are not substantially confounded by external environmental factors. In analyses reported here, we found that the number of medical conditions was significantly positively associated with depressive symptom severity. This aligns with established literature showing that having one medical condition increases depressive symptom prevalence (75–80) and that having multiple medical conditions further compounds this risk (29–32). In this study, we did not distinguish between more and less severe medical conditions and instead summed the number of medical conditions to yield a graded variable. Nonetheless, the sum of all attested medical conditions did not reduce the observed associations between body temperature and depressive symptom severity. These observations suggest that body temperature and one’s burden of medical conditions may contribute independently to depressive symptom severity.

There are several limitations of this study worth noting. First, we assessed depressive symptom severity using PROMIS depression assessments rather than diagnostic evaluations administered by a clinician; therefore, our findings may not generalize to individuals with clinically diagnosed depressive disorders or other psychiatric conditions in which depressive symptoms may occur. Future studies incorporating diagnostic assessments are required to assess whether these associations extend to clinical populations. Additionally, depressive symptoms were assessed monthly, whereas body temperature was measured daily (self-reported data) or continuously (sensor-assessed data). Accordingly, we focused on clinically interpretable, aggregated summary temperature metrics aligned with each depression assessment rather than modeling minute-level temperature trajectories. We did not evaluate additional features of distal body temperature rhythmicity, including intradaily variability and interdaily stability. Future studies should determine whether these nonparametric circadian metrics provide complementary information about associations between distal body temperature rhythms and depressive symptoms. Although we used repeated PROMIS assessments in longitudinal linear mixed models, our primary characterization of depressive symptom severity relied on averaging multiple PROMIS assessments across time. This averaging approach may have reduced sensitivity to shorter episodes or temporal fluctuations in depressive symptoms. Although hypnogram data were used to distinguish asleep and awake periods, we did not include sleep timing in the primary models. Sleep timing may influence distal body temperature patterns and may also be associated with depressive symptoms; however, whether it acts as a confounder or reflects a related aspect of circadian regulation remains uncertain. Future longitudinal studies using explicitly specified causal models and more fine-grained mixed-effects, cosinor, or other circadian modeling approaches should clarify the relationships among sleep timing, body temperature regulation, and depressive symptoms.

Self-reported body temperature measurements were collected using participants’ own thermometers, which may have differed in device type, measurement method, and accuracy. Although this approach enabled assessment of body temperature in a large, geographically diverse cohort, variability in measurement procedures may have introduced measurement error. Similarly, the approximate geographical location at which participants completed the daily survey was considered their physical location for the day. This location may not be precisely representative of the environment in which they spent most of the day. The ability to map ambient temperature to locations was constrained to data available from weather stations, which was limited for individuals living in more remote areas. Future studies incorporating standardized temperature measurements, more granular location tracking, or wearable environmental sensors may enable more precise evaluation of the association between environmental factors, body temperature, and depressive symptoms.

Depressive symptom severity groups were not evenly distributed, with fewer participants reporting more severe depressive symptoms compared with those in milder depressive symptom categories. Although metrics such as ROC performance are determined primarily by separation between groups rather than group size alone, the smaller number of participants with severe symptoms may result in less precise estimates, which should be considered when interpreting classification analyses. Finally, the limited data collection period did not collect data during the winter months in the Northern Hemisphere (where most participants were located during participation (21)). Future longitudinal studies with data collection spanning across all seasons would better capture the full range of environmental variability that may influence depressive symptom severity. Moreover, longitudinal studies extending past the initial years of the COVID-19 pandemic may help disentangle the effects of environmental variables, such as ambient temperature and daylight length, from pandemic-related disruptions to depressive symptomatology.

Our findings reaffirm and expand upon associations between body temperature and depressive symptom severity by incorporating several relevant covariates. Future research should explore the potential of body temperature monitoring as a tool for detecting the onset of depressive episodes and identifying optimal treatment windows.

## Data Availability

The data analyzed in this study is subject to the following licenses/restrictions: Oura’s data use policy does not permit us to make wearable device data (collected via the Oura Ring) available to third parties. Access to anonymized and privacy-protected data may be granted to a qualified academic investigator upon completing agreements with Oura Health Oy and the investigators. Requests to access these datasets should be directed to Ouraring Inc., request_data_access@ouraring.com. Further inquiries can be directed to the corresponding author.
