# Supplementary material for: Body temperature correlates with depressive symptom severity: analysis and extension of prior findings in observational cohorts with more than 19,000 participants
Source: Front Psychiatry. 2026 Aug 19;17:1846398. doi: 10.3389/fpsyt.2026.1846398 (PMC13535770; doi:10.3389/fpsyt.2026.1846398)
Supplement: Supplementary file 1 [file Image1.pdf]

Supplementary Material

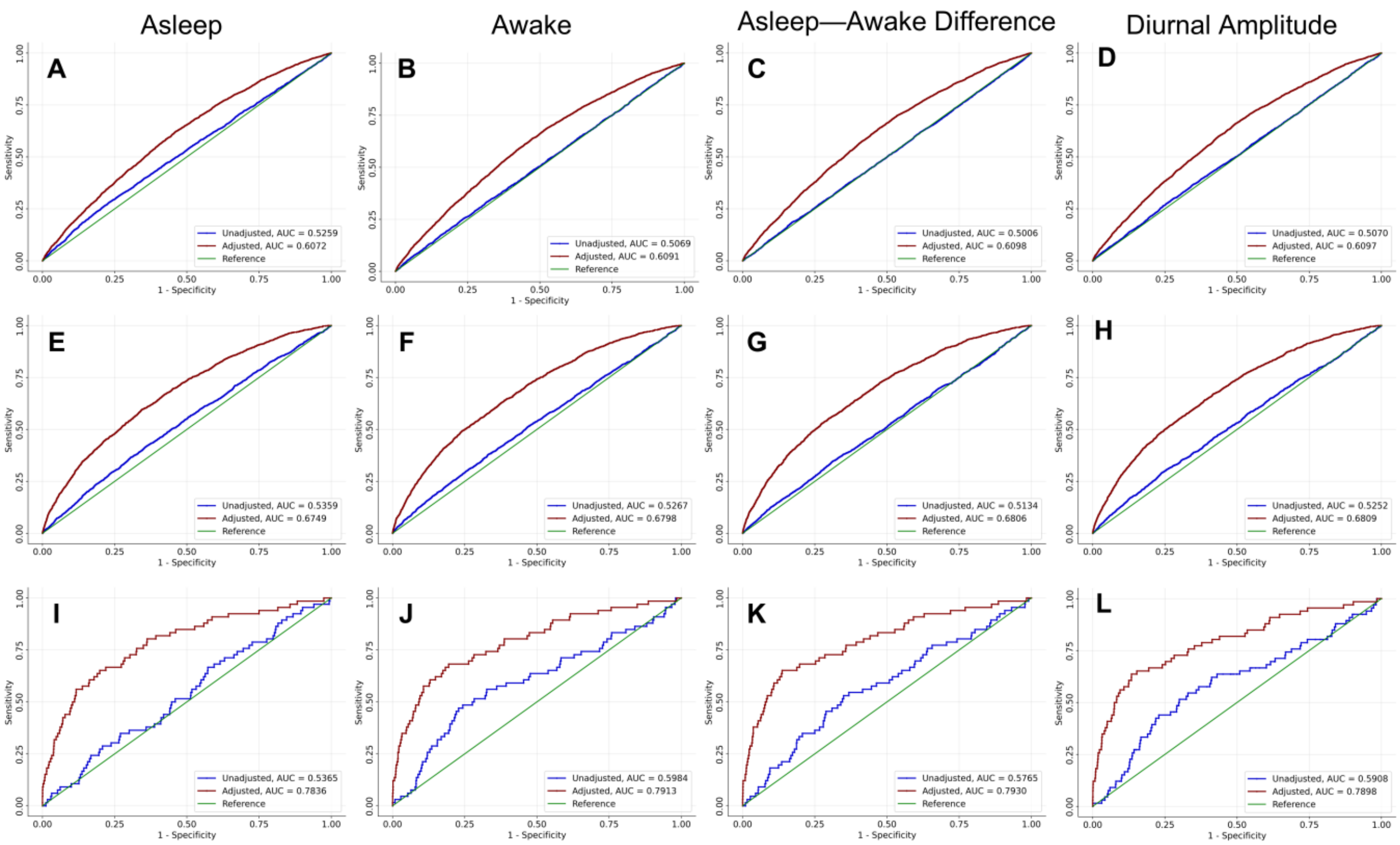

Supplementary Figure 1. Receiver operating characteristic (ROC) analyses for sensor-assessed distal body temperature metrics. ROC curves based on unadjusted and adjusted logistic regression models for PROMIS depression T-scores in the mild range vs. PROMIS depression T-scores within normal limits (WNL) for **(A)** asleep distal body temperature, **(B)** awake distal body temperature, **(C)** asleep-awake distal body temperature difference, and **(D)** diurnal distal body temperature amplitude. ROC curves for the same distal body temperature metrics are depicted in figure panels **(E-H)** for PROMIS depression T-scores in the moderate range vs. PROMIS depression T-scores WNL and in figure panels **(I-L)** for PROMIS depression T-scores in the severe range vs. PROMIS depression T-scores WNL.
