# Supplementary material for: Body temperature correlates with depressive symptom severity: analysis and extension of prior findings in observational cohorts with more than 19,000 participants
Source: Front Psychiatry. 2026 Aug 19;17:1846398. doi: 10.3389/fpsyt.2026.1846398 (PMC13535770; doi:10.3389/fpsyt.2026.1846398)
Supplement: Supplementary file 2 [file Table1.docx]

Supplementary Material

| **Outcome** | **Variable** | **Temperature Cut Point (°C)** | **Sensitivity** | **Specificity** | **ROC AUC** |
| --- | --- | --- | --- | --- | --- |
| **Mild vs. WNL** | Self-reported body temperature | 36.532 | 53.29% | 52.93% | 0.538 |
|  | Asleep distal body temperature | 35.560 | 28.08% | 77.00% | 0.526 |
|  | Awake distal body temperature | 33.156 | 25.16% | 76.63% | 0.507 |
|  | Asleep–awake distal body temperature difference | 4.658 | 18.45% | 82.99% | 0.501 |
|  | Diurnal distal body temperature amplitude | 1.800 | 26.50% | 75.60% | 0.507 |
| **Moderate vs. WNL** | Self-reported body temperature | 36.624 | 45.99% | 63.57% | 0.560 |
|  | Asleep distal body temperature | 35.469 | 36.57% | 69.47% | 0.536 |
|  | Awake distal body temperature | 32.814 | 37.90% | 66.91% | 0.527 |
|  | Asleep–awake distal body temperature difference | -0.833 | 34.81% | 68.71% | 0.513 |
|  | Diurnal distal body temperature amplitude | 1.800 | 29.09% | 76.17% | 0.525 |
| **Severe vs. WNL** | Self-reported body temperature | 36.612 | 61.54% | 62.48% | 0.643 |
|  | Asleep distal body temperature | 35.175 | 66.67% | 42.73% | 0.537 |
|  | Awake distal body temperature | 33.124 | 48.48% | 75.68% | 0.598 |
|  | Asleep–awake distal body temperature difference | -0.833 | 53.03% | 65.04% | 0.577 |
|  | Diurnal distal body temperature amplitude | 1.800 | 54.55% | 67.02% | 0.591 |

**Supplementary Table 1.** Receiver operating characteristic (ROC) curve analyses for each unadjusted logistic regression model predicting PROMIS depression T-score categories (mild, moderate, and severe) versus PROMIS depression T-scores within normal limits (WNL) from self-reported body temperature and wearable sensor-assessed body temperature metrics.

AUC, area under the curve.
