# Supplementary material for: Body temperature correlates with depressive symptom severity: analysis and extension of prior findings in observational cohorts with more than 19,000 participants
Source: Front Psychiatry. 2026 Aug 19;17:1846398. doi: 10.3389/fpsyt.2026.1846398 (PMC13535770; doi:10.3389/fpsyt.2026.1846398)
Supplement: Supplementary file 3 [file Table2.docx]

Supplementary Material

**Supplementary Table 2.** Receiver operating characteristic (ROC) curve analyses for each adjusted logistic regression model predicting PROMIS depression T-score categories (mild, moderate, and severe) versus PROMIS depression T-scores within normal limits (WNL) from self-reported body temperature and wearable sensor-assessed body temperature metrics.

| **Outcome** | **Variable** | **Temperature Cut Point (°C)** | **Sensitivity** | **Specificity** | **ROC AUC** |
| --- | --- | --- | --- | --- | --- |
| **Mild vs. WNL** | Self-reported body temperature | 35.500 | 62.53% | 54.02% | 0.613 |
|  | Asleep distal body temperature | 31.764 | 62.24% | 53.90% | 0.607 |
|  | Awake distal body temperature | 25.222 | 63.73% | 52.73% | 0.609 |
|  | Asleep–awake distal body temperature difference | -0.694 | 64.10% | 52.40% | 0.610 |
|  | Diurnal distal body temperature amplitude | 1.914 | 66.01% | 50.61% | 0.610 |
| **Moderate vs. WNL** | Self-reported body temperature | 35.500 | 62.14% | 63.65% | 0.680 |
|  | Asleep distal body temperature | 31.764 | 59.65% | 65.53% | 0.675 |
|  | Awake distal body temperature | 24.907 | 64.04% | 61.23% | 0.680 |
|  | Asleep–awake distal body temperature difference | -0.655 | 52.91% | 72.61% | 0.681 |
|  | Diurnal distal body temperature amplitude | 1.914 | 64.22% | 61.19% | 0.681 |
| **Severe vs. WNL** | Self-reported body temperature | 35.500 | 75.82% | 68.77% | 0.778 |
|  | Asleep distal body temperature | 32.375 | 65.15% | 80.19% | 0.784 |
|  | Awake distal body temperature | 26.015 | 68.18% | 80.57% | 0.791 |
|  | Asleep–awake distal body temperature difference | -0.655 | 65.15% | 86.34% | 0.793 |
|  | Diurnal distal body temperature amplitude | 1.966 | 63.64% | 86.58% | 0.790 |

AUC, area under the curve.
