# Supplementary material for: Body temperature correlates with depressive symptom severity: analysis and extension of prior findings in observational cohorts with more than 19,000 participants
Source: Front Psychiatry. 2026 Aug 19;17:1846398. doi: 10.3389/fpsyt.2026.1846398 (PMC13535770; doi:10.3389/fpsyt.2026.1846398)
Supplement: Supplementary file 4 [file Table3.docx]

Supplementary Material

**Supplementary Table 3.** Adjusted linear models (incorporating sensor-assessed body temperature metrics) regressing average PROMIS depression T-scores onto ambient temperature, daylight length, and number of medical conditions.

| **Variable** |  | **Model 1:**  **Asleep distal body temp.**  **Regression Coefficient**  **[95% CI]** | **Model 2:**  **Awake distal body temp.**  **Regression Coefficient**  **[95% CI]** | **Model 3:**  **Asleep–awake distal body temp. diff.**  **Regression Coefficient**  **[95% CI]** | **Model 4:**  **Diurnal distal body temp. amp.**  **Regression Coefficient**  **[95% CI]** |
| --- | --- | --- | --- | --- | --- |
| Daylight length (h) |  | -0.149***  [-0.224, -0.075] | -0.216***  [-0.291, -0.141] | -0.210***  [-0.285, -0.135] | -0.214***  [-0.289, -0.139] |
| Ambient temperature (°C) |  | -0.004  [-0.024, 0.016] | -0.018  [-0.038, 0.002] | -0.022*  [-0.042, -0.001] | -0.023*  [-0.043, -0.002] |
| No medical conditions^R^ |  |  |  |  |  |
| Medical conditions not reported |  | -0.287*  [-0.570, -0.004] | -0.179  [-0.462, 0.105] | -0.187  [-0.470, 0.096] | -0.165  [-0.448, 0.119] |
| 1 medical condition |  | 1.084***  [0.647, 1.521] | 1.078***  [0.641, 1.514] | 1.055***  [0.618, 1.491] | 1.044***  [0.607, 1.480] |
| 2 medical conditions |  | 1.623***  [0.964, 2.282] | 1.614***  [0.956, 2.271] | 1.570***  [0.912, 2.228] | 1.554***  [0.896, 2.212] |
| 3+ medical conditions |  | 1.853***  [0.861, 2.846] | 1.867***  [0.877, 2.856] | 1.794***  [0.804, 2.784] | 1.756***  [0.766, 2.746] |

CI, confidence interval; ^R^, reference group. **p* < 0.05, ***p* < 0.01, ****p* < 0.001. Table 1 in the main text shows regression coefficients and 95% CIs from models regressing average PROMIS depression T-scores onto each of the sensor-assessed body temperature metrics described in the table header.
