# Supplementary material for: Body temperature correlates with depressive symptom severity: analysis and extension of prior findings in observational cohorts with more than 19,000 participants
Source: Front Psychiatry. 2026 Aug 19;17:1846398. doi: 10.3389/fpsyt.2026.1846398 (PMC13535770; doi:10.3389/fpsyt.2026.1846398)
Supplement: Supplementary file 5 [file Table4.docx]

Supplementary Material

**Supplementary Table 4.** Adjusted linear mixed models (incorporating sensor-assessed body temperature metrics) regressing monthly PROMIS depression T-scores onto ambient temperature and daylight length.

| **Variable** |  | **Model 1:**  **Asleep distal body temp.**  **Regression Coefficient**  **[95% CI]** | **Model 2:**  **Awake distal body temp.**  **Regression Coefficient**  **[95% CI]** | **Model 3:**  **Asleep–awake distal body temp. diff.**  **Regression Coefficient**  **[95% CI]** | **Model 4:**  **Diurnal distal body temp. amp.**  **Regression Coefficient**  **[95% CI]** |
| --- | --- | --- | --- | --- | --- |
| Daylight length (h) |  | 0.065***  [0.041, 0.088] | 0.049***  [0.026, 0.073] | 0.052***  [0.028, 0.075] | 0.052***  [0.029, 0.076] |
| Ambient temperature (°C) |  | -0.079***  [-0.087, -0.072] | -0.090***  [-0.098, -0.082] | -0.091***  [-0.100, -0.083] | -0.091***  [-0.100, -0.083] |

CI, confidence interval; ****p* < 0.001. Table 4 in the main text shows regression coefficients and 95% CIs from models regressing monthly PROMIS depression T-scores onto each of the sensor-assessed body temperature metrics described in the table header.
